# Return to work experience on structural constraints and gendered differences among nasopharyngeal carcinoma survivors: a qualitative study

**DOI:** 10.1007/s00520-026-11104-z

**Published:** 2026-08-18

**Authors:** Xiaohui Su, Ziqi Peng

**Affiliations:** 1 School of Sociology and Anthropology, Sun Yat-Sen University, Guangzhou, China; 2 Department of Social Work, Hong Kong Baptist University, Hong Kong SAR, China

**Keywords:** Cancer survivors, Return to work, Qualitative research, Nasopharyngeal carcinoma, Psychosocial oncology

## Abstract

**Purpose:**

Working-age nasopharyngeal carcinoma (NPC) survivors frequently resume employment while contending with weak labor protections and latent gender norms. This study aims to explore how NPC survivors navigate the return to work (RTW) process beyond binary aspects, with a focus on the dynamics of structural constraints and gendered experiences.

**Methods:**

This study employed a qualitative design, conducting semistructured interviews with 32 working-age NPC survivors in China (aged 30 to 45 years, 56.2% male). Data were analyzed with thematic analysis.

**Results:**

The findings reveal two overarching themes, with six subthemes. (1) Structural constraints: NPC survivors are encountering reduced sick leave pay, disruption of social insurance, and housing insurance. A cancer diagnosis puts pressure on working performance, but there is limited support for remote work and opportunities for promotion. The coping strategies include optimizing insurance reimbursement with commercial insurance, crowdfunding, applying for leaves, and welfare schemes. (2) Gendered coping strategies on RTW: NPC survivors tend to limit disclosure of their diagnosis to avoid labeling and stigma. Male survivors consistently emphasized breadwinner-related financial toxicity, while female survivors more frequently reported role conflict, indicating a paradox of caregiving and RTW.

**Conclusion:**

The RTW trajectory of NPC survivors is a complex and multifaceted process, navigating multiple significant challenges that are deeply entrenched in structural constraints and gendered expectations, resulting in a dynamic phenomenon. The existing coping strategies might not fully address the identified challenges. Institutional reform should involve standardized sick leave pay, ensuring continuity of social insurance, antistigma practices, and providing support for flexible working arrangements.

## Introduction

Nasopharyngeal carcinoma (NPC) frequently occurs in the southern region of China [1]. Working-age adults (aged 16-64 years) frequently serve as the primary breadwinners of a family [2]. Consequently, the return to work (RTW) journey for this population is faced with psychosocial challenges, including pressure and financial toxicity [3, 4]. Successful RTW is not only a crucial symbol of successful recovery and social reintegration [5] but also benefits the survivors’ self-identity and psychological well-being and mitigates the financial precarity of their families [6]. However, medical treatment, such as radiotherapy-related late effects, including speech impairments, fatigue, and body image changes, hinders the vocational and social reintegration of NPC survivors [7–9]. Empirical studies indicated that 8 to 40% of NPC survivors encounter substantial difficulties in achieving their RTW [4].

In the context of population aging in China, the Departments of Human Resources and Social Security have announced a gradual delay of the retirement age, intending to postpone retirement for males from 60 to 65 years old and for females from 55 to 60 years old [10]. This policy shift carries implications for working-age cancer survivors. For NPC survivors, who are typically diagnosed between ages 30 and 50 and face a protracted recovery trajectory, the extended working life means they must sustain employment for longer periods while managing chronic late effects of treatment [11]. Moreover, the delayed retirement policy intensifies the structural expectation of continuous labor force participation, leaving less possibility for withdrawal, career interruption, or reduced work capacity. This makes understanding of working-age NPC survivors’ navigation of the RTW process under these changes, and their potential gender differences, significant.

Existing studies associate cancer survivorship with employment difficulties and increased unemployment. Specifically, de Boer et al. [12] argued that there is a higher risk of unemployment among cancer survivors than in the healthy population. Furthermore, Yao et al. [13] demonstrated that the majority of Chinese cancer survivors perceived a significant impediment to their work capacity due to the effects of cancer treatment. Compounding the global trend of regional labor intensity and overtime working, particularly in the Asia Pacific region (i.e., exceeding 48 h per week) [14], in this context, the RTW process becomes more challenging for cancer survivors. Samples from mixed urban-rural regions in China illustrated these specific employment shocks. Su et al. [15] reported that within a rural setting, approximately 40% of cancer survivors had ceased working within 1 year post-treatment.

Studies on employment outcomes among cancer survivors in China are predominantly quantitative, examining RTW rates, sick leave duration, or predictors of employment status [16, 17]. These studies tend to frame RTW as a binary outcome, categorizing survivors as either employed or unemployed, thereby obscuring the nuances of vocational reintegration. However, RTW in contemporary society is no longer a dichotomous event but a developmental process involving multiple phases, partial participation, role renegotiation, and ongoing adjustment [18]. This binary framing fails to capture the continuum of work engagement that characterizes cancer survivors’ employment trajectories, and it overlooks how structural constraints interact with gendered expectations to shape distinct pathways through this continuum [5]. An in-depth qualitative examination is therefore needed to reveal the complexities of sociocultural and institutional factors in China, including population aging, labor regulations, and gender differences, thereby providing insights for policy and caring practices. This qualitative study aims to investigate how NPC survivors in China negotiate their RTW experiences in the face of these structural constraints and prevailing gendered expectations.

## Methods

### Study design

A qualitative design was adopted to examine the RTW experiences among working-age NPC survivors [19]. A total of 32 semistructured interviews were conducted in the department of nasopharyngeal oncology at a regional top-tier^1^ cancer center in southern China. The semistructured interview enables the flexible, in-depth probing of individual experiences while maintaining methodological comparability and thematic consistency across the dataset [20]. The reporting of this study followed the Standards for Reporting Qualitative Research (SRQR) to ensure transparency and methodological rigor.

### Participants

The recruitment of NPC survivors and the inclusion and exclusion criteria were designed with two major considerations. First, NPC is one of the most prevalent cancers among young adults (YAs) in southern China, which drives the context-specific recruitment [21]. Epidemiological evidence indicates a substantially higher incidence rate of NPC among the population aged 20 to 49 in this region compared to most other cancers [11]. Second, studies indicate a high RTW rate, with one-third of NPC survivors successfully resuming their work during the early post-treatment period [22]. Therefore, this study focused on the RTW experiences within the specific cohort of NPC survivors, which offers broader and critical insights into challenges encountered by working-age cancer survivors.

Eligible NPC survivors were invited based on the following inclusion criteria: (a) aged 18 years or above, (b) diagnosed with NPC, (c) had returned to work after treatment with at least 1 year of experience, and (d) willing to disclose their personal experience. The exclusion criteria included (a) NPC survivor not in work before or after NPC diagnosis or treatment.

A total of 32 NPC survivors were recruited, comprising 18 males and 14 females, aged 30 to 45 years, across various stages of cancer diagnosis (see Table 1). Eleven participants (34.4%) were employed in government or public institutions or state-owned enterprises. Fourteen of them held positions in private companies, four operated their own businesses as self-employed, and three were temporarily employed. The duration of survivorship ranged from 1 to 15 years, enabling exploration of longitudinal RTW experiences. Given the broader time period, the potential limitation of this sample is acknowledged in the following section.

**Table 1:** Characteristics of the NPC survivors interviewed (*n* = 32)

Characteristics	*n* (%)
**Age group**	
30–35	7 (21.9%)
36–40	18 (56.2%)
41–45	7 (21.9%)
**Gender**	
Male	18 (56.2%)
Female	14 (43.8%)
**Metastatic or recurrence status**	
Metastatic or recurrence	9 (28.1%)
Nonmetastatic or recurrence	23 (71.9%)
**Employment status (current)**	
Yes	19 (59.4%)
No	13 (40.6%)
**Work before cancer diagnosis**	
Government/public institutions/state-owned enterprises	11 (34.4%)
Private enterprises	14 (43.8%)
Self-employment	4 (12.5%)
Temporary employment	3 (9.3%)

Participants were recruited from December 2024 to March 2025. The first author accompanied the attending physician on rounds twice a week in the department of nasopharyngeal oncology to identify eligible NPC survivors meeting the inclusion criteria. Potential participants were first informed about this study either by the attending physician during ward rounds or by a patient support coordinator. The first author approached potential participants only after this initial introduction and only if they had expressed interest in receiving further information. NPC survivors who agreed to participate in this study were contacted by the first author, informed of the study details via WeChat, and scheduled for an interview. All participants noted their participation was voluntary and that refusal would not affect their treatment. The potential participants who declined stated that their decisions were due to time constraints, emotional concerns, or unwillingness to discuss their illness and work experiences.

### Data collection

Each participant was invited to online, one-to-one interviews conducted through the Tencent Meeting platform in a private setting for ethical considerations. The semistructured questions are designed and refer to the study on RTW of cancer survivors [23]. The sample question includes “Can you tell us about the circumstances in which you returned to work after being diagnosed with cancer?” “After returning to work, what constraints did you encounter in recruitment, promotion, or job assignment?” “After diagnosis, how did household income and the division of labor change, and how did these shifts affect your role experiences at home and at work?” Follow-up questions were tailored to participants’ narratives, and interviewers employed empathetic, nonjudgmental facilitation to encourage in-depth reflection. Each interview lasted approximately 60 to 90 min. Participants were explicitly informed that the interviews constituted research activities rather than counseling or therapeutic intervention.

Interviews reached saturation at 25 interviews, and an additional 7 interviews were conducted for data trustworthiness [24]. Specifically, this strategy served three purposes as follows. First, this strategy verified saturation through testing whether new coding and themes contradicted established patterns. Second, this strategy ensures the confirmability of the findings with the additional cases. Third, this strategy enriches the representation of underrepresented demographic subgroups, including participants with recurrence or metastasis and self-employed individuals, thereby providing a comprehensive thematic framework.

All interviews were audio-recorded and transcribed verbatim for data analysis. All transcripts were pseudonymized to rigorously protect participants’ identities, and the first author meticulously cross-checked them for accuracy. The transcripts were then returned to all 32 participants for member checking via WeChat. Participants were given 2 weeks to review and amend any content they considered inaccurate. Twenty-six participants responded within the designated period. Most participants presented confirmatory statements, with several making minor corrections to the dates or job titles. No participant contested the content of their interviews. A follow-up reminder was sent to those who did not respond. Participants who remained unresponsive were treated as having no objections in accordance with the informed consent agreement.

### Data analysis

Thematic analysis was applied for data analysis [25]. The interview data was preprocessed and analyzed by the first and second authors through MAXQDA (version 24.11.0). MAXQDA is a robust qualitative analysis software that enables collaborative coding, analysis, and visualization of qualitative data.

The analysis employed a three-step procedure to assess data trustworthiness. First, two authors independently performed the coding process, an iterative process designed to gain a deeper understanding of the qualitative data. They subsequently employed open coding to assign preliminary descriptive codes to the textual data. Second, two authors generated codes through reviewing and comparing the generalized themes, ensuring the consolidation of the coding framework. Third, cross-checking was performed to resolve disagreements until a consensus was reached by two authors. Two overarching themes and six subthemes collectively represented the distinct challenges encountered by the interviewees across the RTW trajectory (see Table 2).

**Table 2:** Identified themes and subthemes

Theme	Subtheme
Structural constraints	1. Absence of institutional supports
	2. Being marginalized in the labor market
	3. Mobilized resources for coping
Gendered coping strategies on return to work	1. Gendered patterns on coping with stigma
	2. Gendered patterns on financial toxicity
	3. Gendered path of employment trajectories and work withdrawal

## Results

### Structural constraints

Both male and female participants consistently identified a shared RTW dilemma, the insufficient institutional support for cancer survivors, including continued exposure to rigid attendance policies, performance pressures, and a lack of remote work support.

#### Absence of institutional supports

NPC survivors’ RTW under institutional and economic challenges. While cancer-related fatigue sufferers, the performance and attendance requirements remained. During their sick leave, social insurance and housing insurance payments were transferred to them, along with a reduced salary. Some participants reported that the prolonged time required for cancer treatment and recovery caused them to exceed the statutory age limits for national recruitment examinations, effectively foreclosing career advancement pathways that would otherwise have been available. This temporal displacement reinforced their vulnerability and constrained equality of opportunity in the RTW process.

*“Before I got sick, my position allowed more flexible hours. In the new role, I have to clock in and out, and there’s less freedom.” (Male, public institution employee, aged 45)*

*“I took seven months of sick leave and had no income during that time. I even had to pay my own social insurance and housing fund. Without my colleagues’ fundraising, I wouldn’t have made it through.” (Male, private enterprise employee, aged 38)*

*“I was 31 last year. I planned to take the adult higher-education entrance exam, enroll in a three-year program, and, after graduation, sit for the staffing exam at a new hospital near my home to secure a stable civil-service position. I had already registered for the October exam, but a cancer diagnosis and treatment derailed everything. Even if I retake it this year, by the time I obtain the degree certificate, I will be over the 35-year age limit and will have lost my only chance to sit the staffing exam.” (Female, private enterprise employee, aged 32)*

#### Being marginalized in the labor market

In the post-treatment stage, participants demonstrated a strong desire to RTW. Specifically, they often shorten sick leave in order to preserve their career positions and client bases. However, upon re-entry, they found that evaluation and promotion were not only related to working performance but also to perceived “health eligibility.” While their supervisor often expresses sympathy, this did not translate into substantive adjustment. Working performance requirements remained unchanged, failing to accommodate post-treatment limitations. As a result, health status operated as a hidden criterion of employability, narrowing equitable pathways back to work.

*“I’ve been with the company for over a decade. I was entitled to a year of treatment leave. I feared losing my position and dropping from a managerial post. Four or five months before the leave ended, I applied to work from home. My boss agreed readily.” (Female, state-owned enterprise employee, aged 35)*

*“I really pushed myself. I finished treatment on May 12 and only rested at home for a month. By June, I was back at work, wearing a wig.” (Female, private enterprise employee, aged 37)*

*“If I hadn’t gotten sick, I would likely have been promoted to Deputy General Manager by now. After the illness, my abilities didn’t decline, but I no longer meet the health requirements. When the unit selects leaders, they prioritize those who will be reliably ‘usable’ in the future. What I need to do now is to prove that I won’t have health problems for the next three to four years.” (Male, public institution employee, aged 33)*

#### Mobilized resources for coping

Survivors actively mobilized structural resources to cope with the socioeconomic and physiological challenges of long-term recovery. To mitigate the financial toxicity of reduced income related to sick leave, they optimized insurance reimbursement rates and leveraged local health insurance schemes to maximize reimbursement coverage for treatment and living expenses. Furthermore, survivors utilized parental and annual leave to balance work duties and the need for frequent medical consultations. This strategy enabled them to utilize all available institutional flexibility to meet their survivorship needs.

*“I originally had out-of-province health insurance. Later, I found someone to help me purchase the local coverage, which can be bought this month and takes effect next month, and the reimbursement rate is higher than the original one.” (Male, private enterprise employee, aged 35)*

*“I had no clear sense of the treatment costs before starting cancer treatment. I first asked the company to pre-pay 60,000 yuan salary for treatment, but my supervisor declined, claiming there was no precedent. With my consent, my supervisor then organized a staff fundraiser, about a hundred colleagues participated and raised over 50,000 yuan, which largely filled the gap.” (Male, private enterprise employee, aged 32)*

*“I’m still in the breastfeeding period, so it’s fortunate I can apply for parental leave. Otherwise, coming in for follow-up treatment with sick leave would be quite troublesome to apply. If I take annual leave, that day is unpaid.” (Female, public institution employee, aged 37)*

### Gendered coping strategies on return to work

#### Gendered patterns on coping with stigma

In the RTW context, the results identified distinct gendered patterns of coping strategies with stigma. Female participants prioritized control over information and protection of privacy. For example, a survivor disclosed her diagnosis selectively to the deputy human resources director to secure extended leave, returned wearing a wig, and withheld her diagnosis from colleagues to avoid labeling. In contrast, male participants emphasized masculinity maintenance and social withdrawal. This is demonstrated by behaviors such as eating alone (due to misconceptions related to contagion), social withdrawal, or actively avoiding group meals. Others announced their diagnosis through sick leave procedures. Even when receiving colleagues’ support, they reported enduring feelings of inferiority and a persistent burden of holding patient identity.

*“I brought my pathology report to my supervisor when I requested leave and burst into tears; she told me not to think about anything else. After I calmed down, I asked her to keep it confidential and told no coworkers. I returned to work one month after completing treatment, wearing a wig. Many assumed I’d been on maternity leave; they were surprised by my weight loss and how thin I looked, and some were curious because the wig looked unnatural.” (Female, private enterprise employee, aged 37)*

*“I took sick leave through the company system, so I couldn’t keep it fully private. People found out without my saying anything. My relationships barely changed. Friends who used to drink and smoke with me now step outside to smoke. Coworkers look out for me on their own. I still carry the ‘patient’ label in my head, which makes me self-consciouss.” (Male, public institution employee, aged 36)*

*“I felt anxious about returning and unsure how people would see me. Some might avoid meals or conversation for fear that EBV is ‘contagious.’ At first I ate alone in a corner. The fewer people, the better. Even now I am cautious. I join only if others are willing and I try not to be too forward. Some still say that once you have had EBV, you are contagious.” (Male, public institution employee, aged 37)*

#### Gendered patterns on financial toxicity

NPC survivors are confronted with the risks of diagnosis and recurrence. They and their families entered a complex renegotiation of financial issues, family relationships, and self-identity. Reduced income and mortgage pressure forced them to weigh final strategies like insurance, crowdfunding, kinship support, and re-employment. The household labor was reorganized, and female survivors encountered a dual burden of care and breadwinning. Specifically, some survivors are pursuing divorce for self-agency to resist gendered expectations.

*“I was the main earner after my child’s father’s business failed and he returned to low-paid work. My mother-in-law handled the home and childcare. After diagnosis I did not fear death. I feared that no one would keep the family afloat. I even considered asking a trusted friend to marry him for our child’s sake. I returned to full-time work in early 2017. In July I learned of bone metastases. I then had two years with no income. After 2019 I juggled childcare with part-time jobs. In 2021 I went back to full-time work while doing most of the care. My hope now is that my husband can earn enough to buy our son a small home of 40 to 50 square meters so he has a place to settle.” (Female, private enterprise employee, aged 44)*

*“My ex-husband judged a wife by whether she could raise children, run the household, and earn money. When I was diagnosed with nasopharyngeal carcinoma our fraternal twins were just over three. One boy and one girl. They were often ill. I handled almost all of their care. He showed little regard for my effort or exhaustion. Without financial independence I did not dare to leave. If I had the means I would have ended the marriage sooner. I am now forty-four and self-employed. I am no longer afraid. With diligence and commitment I can support my children on my own. I chose to end the marriage.” (Female, self-employment, aged 44)*

However, this renegotiation was gender divided. Male survivors focused on negotiating “face” (i.e., mianzi, specific to Chinese culture, which refers to public self-image, dignity, and reputation). They employed strategies of scaling down consumption, prioritized avoiding debt, and sought to sustain their breadwinner role.

*“I used my treatment and recovery time for study and self-improvement. I have always been a reader. I worked through management texts and MBA materials to sharpen my thinking. I studied nutrition and rehabilitation. I planned whole-food meals with fruits and vegetables and looked for ways to strengthen my immunity. My goal was clear. Even if my health did not improve, the learning would not be wasted. Once I recovered, I would use it at work to raise my income and ease the family burden. The responsibility at home rests on me. I have to keep the family steady.” (Male, private enterprise employee, aged 42)*

*“I valued pride and saving face, so at first I told no one. Later, I weighed pride against my family’s needs and set my ego aside. I launched a ShuiDiChou (a Chinese fundraising platform) crowdfunding campaign. My university classmates rallied and raised over 100,000 yuan (over 14,000 USD), which covered the worst expenses. I set two rules for myself. Take on no external debt. Do not sell the home, even if all my income goes to treatment. I am the first in my family to move from the countryside to the city. If I sell the home, my child may be pushed back to the countryside and lose footing. I cannot let us end up with nothing.” (Male, public institution employee, aged 34)*

Despite these different challenges, shared values emerged. A “career-first” priority was replaced by a “family-first” approach, indicating that NPC survivors were devoting more time to their children.

*“I now give my time at home to my child and family. Before diagnosis I focused on my career. Afterward I felt how easily the present can slip away. I value companionship more. When I am home my time belongs to my child until he falls asleep. I used to check my phone or go play basketball after work. Now I try not to be distracted.” (Male, public institution employee, aged 37)*

#### Gendered path of return to work trajectories and work withdrawal

Women’s career trajectories were primarily characterized by withdrawal and identity reconfiguration. Participants found high-pressure demands, such as overtime work and night shifts, to be untenable. This often led them to negotiate contract termination after recurrence, missed promotion opportunities, or transition to family caregiver. This move from the workplace to the family prompted a reassessment of the value of care and emotional bonding.

*“I had planned to return after maternity leave. During leave I was diagnosed. I informed the company and discussed follow-up arrangements. My supervisor offered comfort and support. After more than a year of treatment and recuperation I prepared to resume work. Metastasis was found. My work was interrupted and I agreed with the company to terminate the contract. I now focus on family and childcare. I am a full-time homemaker. I keep a steady routine and a calm state of mind.” (Female, private enterprise employee, aged 34)*

*“I aim to achieve economic independence and a stable life within one to two years. I will pursue steady progress, ease emotional pressure, and loosen external constraints. I want more time with my family and my child.” (Female, private enterprise employee, aged 37)*

Male participants focused on sustaining their identity as breadwinners through adaptation. Rather than withdrawing, they modified their existing roles by moving to lower-intensity posts, reducing overtime, or taking medical retirement. Others actively pursued new avenues, such as career changes or entrepreneurship, to rebuild their skills and income on a new track.

*“I worked a day job and ran a side business before the illness. After the diagnosis, the company dissolved. I did not stay idle. I looked for a new track. I now have an initial direction and outline. The plan and steps still need work.” (Male, public institution employee, aged 36)*

*“After completing treatment, I started with a street stall and built my own brand. It has some recognition in our county. I had worked in surveillance design. Three years of treatment cut me off from the field, and I lost fluency with the software. The industry moves fast, and the pressure is high. I judged that I could not catch up quickly. I left the profession and focused on the new brand.” (Male, private enterprise employee, aged 42)*

## Discussion

Work serves as a central underpinning in the lives and identities of adult cancer survivors and is embedded within the structure of modern healthcare and social welfare systems. Existing studies indicate that successful RTW can offer multifaceted benefits for survivors, including the mitigation of social isolation and the improvement of self-worth, resuming of daily routine, alleviating severe financial toxicity, and improving quality of life [16, 26–28]. From a broader societal perspective, actively promoting RTW among cancer survivors is essential for reducing social welfare costs by sustaining labor force participation [29].

The findings of RTW experiences of NPC survivors in China illustrate two crucial cross-cutting themes: structural constraints and gendered coping strategies. First, in the notable absence of flexible institutional support with inadequate workplace support, survivors face individualized economic and occupational risks of illness. These results align with studies on Chinese cancer survivors and their families, who often bear substantial financial burdens with needs on borrowing and consumption downshifts [30, 31]. Similarly, studies on employers of survivors indicate that RTW support is challenging within existing regulations, with limited scope for adjustment [32]. Survivors more frequently experienced discrimination in areas such as resignation, promotion, salary, and benefits [33]. Second, we identified gendered coping strategies of RTW: Male survivors commonly pursue occupational shifts to preserve their breadwinner role, which echoes stronger financial and continuity pressures [34]. Female survivors more often oscillate between intensified household financial burdens and work withdrawal, relying greater on information strategies for coping with stigma [35, 36].

The absence of effective institutional protections for cancer survivors is a significant challenge to RTW. Explicit salary loss during sick leave and the shifting of mandatory social insurance and housing insurance contributions entirely onto individual employees constituted institutionalized economic penalties. Aligning with a study on Chinese osteosarcoma survivors, the persistent long-term psychosocial support needs and significant barriers to integrating medical and psychological care underscore systemic gaps that effectively shift the burdens of recovery onto the families’ caregiving [37]. Additionally, Tiedtke et al. [32] demonstrate that, within rigid regulatory and performance evaluation, cancer survivors perceive RTW as both “demanding” and “dilemma-laden,” making initial flexible and reasonable strategies inherently difficult. Although many cancer survivors could significantly benefit from working from home, the potential of such arrangements is constrained by the occupational structure (i.e., the nature of occupation) [38]. This suggests that employers prioritized physical attendance over working capacity, placing survivors at a distinct competitive disadvantage. Confronted by these structural constraints, the vulnerability of cancer survivorship shows as rigid attendance requirements and key performance indicator (KPI) standards, which force survivors into a time-compressed RTW before functional recovery is achieved. In the absence of reasonable remote work policies, attendance is often misrecognized as a proxy for work ability, entrenching inequality. This comes across as a financial toxicity that reduces the quality of life of cancer survivors through delayed medical care for working; in turn, the slow recovery process hinders RTW [39].

Compared with other countries, the USA treats people with cancer as individuals with disabilities. The Americans with Disabilities Act Amendments Act (ADAAA) provides a broad definition of long-term functional impairments and emphasizes the need to address employment-related structural constraints, such as stigma and discrimination for cancer survivors [33]. In Japan, the Ministry of Health, Labor and Welfare has issued workplace guidelines to help employees with cancer balance treatment and work, enabling flexible working hours and sick leave with medical notes [40]. However, in China, the “General Standards for Physical Examinations for Civil Servant Recruitment” impose strict medical criteria on cancer survivors, requiring a certificate of recovery before appointment [41]. This gatekeeping approach treats health as a binary credential rather than a complex, interacting structural, cultural, and political factor. Structurally, China’s segmented welfare regime ties social protections closely to formal employment status and employer type, leaving employees in private enterprises and informal sectors with limited leverage to negotiate accommodations [42–44]. Culturally, the dominant productivist welfare ideology positions health management as a personal responsibility, discouraging survivors from demanding workplace support [45]. Politically, disability policy has prioritized visible physical disabilities over conditions such as cancer survivorship, while competitive pressures facing Chinese enterprises imply resistance to survivor employees, considering the additional labor costs [46, 47]. Compared with the USA and Japan, China lacks policies to recognize cancer survivors’ right to reasonable accommodation. Although NPC 5-year survival rates in China now exceed 80%, no explicit policy supporting cancer survivors’ RTW has yet been implemented [48]. Bridging this gap requires both legal reform and a reconsideration of the relationship between health, productivity, and citizenship in labor policy.

The second structural dilemma is medicalized marginalization in the labor market. Participants’ experiences demonstrate that health is instrumentalized as a metric for measuring work performance. Employers evaluate their employees against pretreatment KPIs and expectations of sustained availability for 3 to 5 years, thereby turning health status into an implicit hiring standard. Prior studies have noted that late effects of cancer and its treatment, such as fatigue and cognitive or attentional deficits, serve as key barriers to RTW and career advancement [49, 50], while limited awareness among employers and colleagues of these late effects amplifies differential treatment and impedes promotion pathways [51, 52]. Qualitative research in China further indicates persistent needs for psychosocial support among cancer survivors and inadequate integration of medical and psychological care, with healthcare professionals facing institutional barriers. These patterns limited the pathway for cancer survivors’ help-seeking on RTW and its implicit exclusionary effects [53]. This highlights the importance of institutional assistance in facilitating RTW transition, including workload and timetable adjustments, for a sustainable model of RTW for cancer survivors [27, 54]. Thus, even when a supervisor expresses sympathy, the rigid assessments (i.e., attendance rather than capacity), the marginalization of cancer survivors will be reproduced.

In the context of limited institutional protections and marginalization, the participants tend to utilize institutional resources to mitigate disadvantage. This aligns with Cui et al. [55], who suggest that more flexible regulation will improve inpatient access and per-visit reimbursed expenditures, shifting from a declining to a rising trend without increasing individual financial burden. This, in turn, suggests that such policies help buffer patient costs [55]. Second, regarding the fundraising channels, cancer-related crowdfunding yields highly heterogeneous outcomes shaped by project and textual features and is associated with social inequality, producing systematic differences across groups [56, 57]. Third, the strategic use of leave within existing institutional boundaries to coordinate follow-up care with work tasks. This reflected the trend that approximately 40% of survivors extended leave or modified their work schedules [58]. Overall, these practices demonstrate a post-diagnosis “leveraged life.” The available pathways are typically proactive, temporary, and fragmented in nature. Cancer survivors depend heavily on individual and family social capital for reintegration. This further underscores the urgent need for work-related interventions for this population, such as customized job positions and workload adjustments and reduction, and graded RTW, rather than shifting responsibility to survivors or their families [59–61].

In the context of limited institutional protections and workplace marginalization, gendered differences in RTW among NPC survivors are evident. Female survivors often carry a dual burden of unpaid care and income generation, which can lead to withdrawal from employment or movement between household responsibilities and labor market participation. Male survivors place greater emphasis on preserving a breadwinner role and a masculine identity, and they commonly adopt proactive strategies tied to work in order to maintain status. These orientations also shape identity management and responses to stigma. Men frequently practice self-concealment and avoid peer sociality, for example, skipping group meals because of contagion fears, to sustain an image of a strong and reliable worker and to resist being classified as a patient, which is consistent with evidence on masculinity reconstruction after treatment [62]. Prevailing misconceptions about cancer intensify workplace avoidance and self-concealment, creating a peer level boundary maintenance mechanism that protects position at work. Women, in contrast, tend to use selective disclosure as a defensive professional strategy, managing the visibility of illness to preserve the image of a competent employee and to reduce the risk of labeling or discrimination when employment consequences are salient [63, 64]. Financial toxicity and health outcomes interact in a reinforcing way, since economic pressure reduces quality of life and is associated with poorer survival, which ultimately amplifies inequalities that follow gendered patterns.

In the context of illness shock and constrained supports, survivors and families enter an ongoing renegotiation of identities and relationships as they work to sustain a daily new normal under embodied limitations and emotional strain while reallocating time, care, and income between intimacy and employment [65]. Illness functions not only as a biomedical disruption but also as a sociological process that reveals existing inequalities. Female participants often shift defensively into full-time caregiving or more flexible employment and in doing so reassess and enact caregiving value, which indicates agency in identity rebuilding and decision-making [66, 67]. Male participants place greater emphasis on preserving a breadwinner identity by cutting consumption, avoiding debt, retaining the home, and using retraining and occupational shifts to restore or enhance household economic security [68]. Even with physical decline, men tend to pursue lateral moves rather than exit employment, for example, transferring to less strenuous positions or starting side jobs and small businesses, which operates as a key mechanism for maintaining a work-linked social identity [69]. At the same time, they reallocate limited energy toward time with and care for children, a contemporary expression of caring masculinities that does not fundamentally unsettle the breadwinner role [70]. These patterns highlight how illness compels survivors to construct a new normal and how gendered expectations shape both the risks and the forms of post-diagnosis career trajectories.

## Implications for cancer survivors

In the context of limited institutional protections and growing chronicity in the workforce, smoothing the RTW for cancer survivors requires coordinated action across policy and workplace practice. Policy should establish enforceable safeguards against discrimination and economic penalties, provide income protection through treatment and recovery, and align health and labor regulations to support employment continuity. At the workplace, organizations can reform rigid attendance metrics with capacity-based assessments and reasonable accommodations, including graded RTW plans, flexible scheduling, task redesign, and temporary workload reduction. Gender-sensitive supports are needed to address the distinct pressures faced by men who seek to preserve a breadwinner identity and women who balance caregiving with income generation. Social education should be employed to deconstruct misconceptions about cancer to reduce stigma, self-withdrawal, and social avoidance. Building an inclusive labor market advances equity for survivors and also strengthens societal resilience as more working-age adults manage long-term cancer survivorship.

## Limitation

This study sheds light on how structural constraints and gendered dynamics influence the RTW process among NPC survivors. Findings from 59.4% of employed participants limit insight into job loss and reintegration under health certificate requirements that can lead to medicalized exclusion. The cross-sectional, single-interview design prevents the tracking of within-person change, and single-center recruitment in Guangzhou limits the transferability to less developed and rural settings, where work is more physically demanding. Additionally, although the gender distribution of our sample (56.2% male, 43.8% female) roughly reflects the epidemiological profile of NPC in China [1, 11], the relatively smaller number of female participants may not fully capture the diversity of women’s RTW experiences across employment sectors and caregiving configurations. Future research employing purposively stratified or gender-balanced sampling designs could further validate and refine the gendered patterns identified in this study. Future research should include unemployed survivors, use longitudinal and multisite designs, and examine hiring standards and health certificate rules to address structural barriers.

## Conclusion

This study revealed cancer primarily as a catalyst, intensifying the gendered inequalities already embedded in household divisions of labor, labor markets, and cultural expectations, rather than creating new forms of inequality. Although the findings demonstrate how existing gender norms channel men toward maintaining the breadwinner role and women toward care-oriented withdrawal, this interpretive argument limits the phenomenon in ways that might not articulate whether cancer merely amplifies pre-existing inequalities or simultaneously produces new forms of disadvantage. For instance, the intersection of cancer stigma and gendered employment barriers, such as the compounding effects of health certification requirements and age-restricted civil service examinations, may constitute novel forms of structural exclusion that did not exist prior to diagnosis. Therefore, cancer primarily serves to amplify and make visible existing inequalities, while it may simultaneously play a role in producing new psychosocial challenges. The experiences of NPC survivors offer a lens, revealing how persistent gender orders shape personal life trajectories and cancer survivorship. Women are pushed toward caregiving roles, while men are required to continue playing breadwinning roles through performances of resilience. The reproduction of gender inequality amid the cancer crisis should be further examined through longitudinal research to clarify the relationship between amplification and the production of inequality in the sociocultural contexts.

## Data Availability

The datasets generated during and/or analyzed during the current study are not available due to ethical considerations.

## Footnotes

1 “Sanjia Hospital (class three, type A)” refers to the highest tier of hospital classification in mainland China.
